# Assessment of the antibacterial effect of Barium Titanate nanoparticles against *Staphylococcus epidermidis* adhesion after addition to maxillofacial silicone

**DOI:** 10.12688/f1000research.132727.2

**Published:** 2023-11-06

**Authors:** Yasir Mohammed Kareem, Thekra Ismael Hamad

**Affiliations:** 1 B.D.S. Department of Prosthodontic, College of Dentistry, University of Baghdad, Baghdad, Baghdad Governorate, Iraq; 2 B.D.S., M.Sc., Ph.D., Prof. Department of Prosthodontic, College of Dentistry, University of Baghdad, Baghdad, Baghdad Governorate, Iraq

**Keywords:** barium titanate (BaTiO3), VST-50 silicone elastomer, bacterial adhesion, Staphylococcus epidermidis.

## Abstract

**Background:** Maxillofacial silicones are the most popular and acceptable material for making maxillofacial prostheses, but they are not perfect in every sense. To enhance their effectiveness, more improvements to their properties are required, such as their antimicrobial efficiency.

This study assess the antibacterial effect of barium titanate nanoparticles in various percentages against *staphylococcus epidermidis* biofilm adhesion after addition to maxillofacial silicone.

**Methods:** Barium titanate nanoparticles were added into VST-50 platinum silicone elastomer in four weight percentages (0.25wt%, 0.5wt%, 0.75wt% and 1wt%). 50 specimens were prepared and categorized into five groups; one control group and four experimental groups. All conducted data was statistically analyzed using (one-way ANOVA) analysis of variance, and Games-Howell multiple comparison test (significant level at *p* *<* 0.05). Shapiro-Wilk and Levene’s tests were used, respectively, to evaluate the normal distribution and homogeneity of the data.

**Result:** One-way ANOVA test revealed a highly significant difference between all groups, and Games-Howell test revealed a highly significant difference between the control group and the four experimental groups. The 0.25wt% and 0.5wt% groups revealed a highly significant difference between them and with the (0.75%wt and 0.1%wt) groups. While the 0.75wt% group revealed a significant difference with 1wt% group.

**Conclusions:** The addition of barium titanate to VST-50 maxillofacial silicone enhanced the antibacterial activity of silicon against *Staphylococcus epidermidis*, and this activity seems to be concentration dependent. FTIR analysis demonstrated no chemical interaction between the Barium Titanate and the VST-50 maxillofacial silicone elastomer. SEM pictures show that the barium titanate nanopowder was effectively dispersed inside the maxillofacial silicone matrix.

## Introduction

The restoration of abnormalities resulting from inherited or acquired causes, such as cancer or trauma, has often utilized prosthetic devices. Due to the location and extent of the lesion, surgery may not always be a solution; hence, the need for prosthetic rehabilitation has grown correspondingly. ^1^

Any abnormality that develops in the body, especially in the head and neck area, has a negative impact on the patient’s attractiveness, function, social acceptability, and psychological confidence. The most vital and difficult part of these individuals’ therapy is rehabilitation. Any rehabilitation process attempts to reintroduce the patient into society in a condition that is close to normal. A facial prosthesis preserves the tissues of a defect, restores normal anatomy and appearance, and offers the patient significant psychological benefits. ^2^

Maxillofacial prosthetics can be made from a variety of materials, such as chlorinated polyethylene, polyvinyl chloride, polyurethanes, polymethyl methacrylate, and polydimethylsiloxane.

Silicone elastomers have become more important in medicine and the construction of maxillofacial prostheses because of their strength, durability, ease of manipulation, esthetics, and flexibility. ^1^^,^^3^ Currently, no facial prosthetic material, including silicone, satisfies all the requirements for a satisfactory prosthesis. The primary cause of routine facial prosthetic replacement is deterioration in appearance caused by changes in physical characteristics and color ^3^ not only the antibacterial effect of BaTiO _3_ is important. The other mechanical and physical properties of BaTiO _3_, such as tear strength, tensile strength, thermal properties, etc., are needed to maintain prosthesis durability. ^4^ Therefore, silicone maxillofacial material requires reinforcement.

During the development of the nanoparticle industry, nanoparticles have been incorporated into the polymer matrix as fillers to provide a modified polymer characterized by improved features gained from the reinforcing action of the nanoparticles. The expected mechanical, physical, and biological properties of a silicone elastomer depend on the type and amount of filler added to the polymer. These little additions could enhance certain characteristics of the material. ^1^

Biofilm formation on the surface of maxillofacial prostheses is one of the most critical problems. Biofilms are formed due to various reasons, such as fungal, bacterial, and commensal microflora. These microbes have a clear association with reports of bacterial dermatitis and endophthalmitis. ^6^ Among the different species found, the most frequent have been *Staphylococcus aureus* and *Staphylococcus epidermidis.* The major limitation of maxillofacial silicone is that it has numerous porosities on its surface that are colonized by these microorganisms. ^5^^,^^6^

*Staphylococcus epidermidis* is the most prevalent commensal bacteria on human skin. Although *S. epidermidis* defends us against foreign invasion, it also takes advantage of human weakness when it has the chance. Such chances appear in immunocompromised people or when biomedical implants provide a chance for surface colonization and biofilm formation. ^7^

The physical rubbing or brushing of maxillofacial prostheses is one method of disinfection, although it is not perfectly advised since the repetitive cleaning might roughen the material’s surface. Similar chemical immersion, for example, repeated use of chlorhexidine gluconate CHX, may change the physical and mechanical characteristics of maxillofacial silicone elastomers, resulting in roughness, color change, and an increase in microhardness. ^6^

Incorporation of a nanoparticle such as Barium Titanate (BaTiO _3_) may enhance the antimicrobial and other properties of maxillofacial silicon. Barium titanate (BaTiO _3_), a dielectric/ferroelectric semiconductor, is the most extensively used photocatalyst in environmental and medical applications due to its low cost, chemical stability, biocompatibility, and non-toxicity. BaTiO _3_ has been proven to accelerate osteogenesis, and the same material in nanoparticle form acts as a second harmonic generation (SHG) probe to identify Osteogenesis Imperfecta. ^8^^,^^9^

BaTiO _3_ had shown antibacterial activity against numerous types of bacteria when added to different materials such as polyvinylsiloxane, hydroxyapatite, and implants. ^10^^,^^26^ This study aimed to evaluate the effect of BaTiO _3_ on *S. epidermidis* biofilm adhesion after addition to VST-50 maxillofacial silicone in various weight percentages.

Hypothesis of study: **The null hypothesis (H0)** suggests that adding BaTiO _**3**_ nanoparticles will not affect bacterial adherence. **The alternative hypothesis (H1)** suggests that adding BaTiO _**3**_ nanoparticles will significantly reduce bacterial adherence.

## Methods

Barium Titanate (BaTiO _3_) (Sky Spring Nanomaterials, USA) and VST-50 room temperature vulcanized silicone (Factor II Inc., USA) were used.

Particle size analyzer was used to verify that the BaTiO _3_ particles are at the nanoscale, and the effective diameter was (59.4 nm).

### Specimen grouping

50 specimens were prepared and categorized equally into five groups: one control group (0wt% BaTiO _3_) and four experimental groups (0.25wt%, 0.5wt%, 0.75wt%, and 1wt% BaTiO _3_) 10 specimens for each group.

### Mold fabrication

Three clear acrylic sheets (the matrix, bottom, and cover) with 2 ± 0.05 mm thickness were created. The matrix sheet was designed with 10 mm disk-shaped perforations and was glued to the bottom sheet by chloroform (glue material) to avoid its moving while silicone was being poured. Using a computer’s software (CorelDraw 2020) to design the mold and a CNC machine to fabricate it. Clamps, screws, and nuts were also used for further tightening at the edges, ^1^^,^^11^ an alternative open-source software is FreeCAD.

### Mixing

In accordance with the manufacturer’s instructions, the VST-50 maxillofacial silicone is mixed at a ratio of 10:1 (10 parts base to 1 part catalyst). A vacuum mixer had been used to prevent air entrapment.

Specimens for the control group were mixed by using an electronic digital balance (to 3 dp) for weighing the base and catalyst, then mixed for 5 minutes.

For the experimental groups, BaTiO _3_ filler was first weighted using digital balance and added to the bowl, followed by the weighing and addition of the base part to the filler. The mixture was mixed for 3 minutes without vacuum to avoid suction of the filler, followed by 7 minutes of mixing with air suction. The vacuum pressure is set to -10 bar (-28 inch Hg), and the speed is set to 140 ± 10 rpm. The mixture was then allowed to cool for 5 minutes. The catalyst was then added to the base-filler mixture and mixed for 5 minutes. ^12^^–^^14^

**Figure 1.**
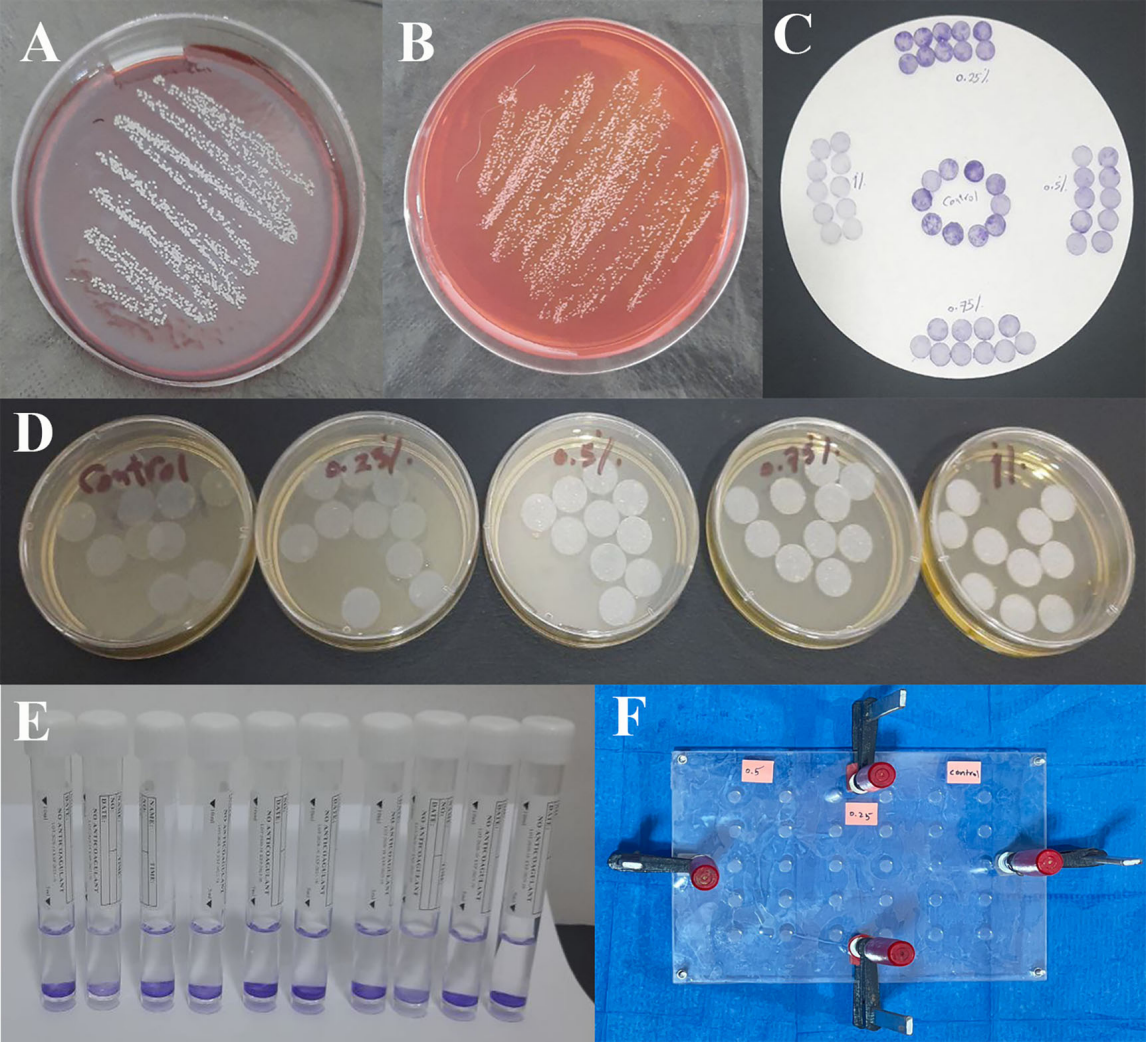
A, *S. epidermidis* incubated on blood agar media; B, *S. epidermidis* incubated on mannitol salt agar media; C, Specimens after staining by crystal violet and rinsing; D, Specimens incubated in bacterial suspension; E, Specimens immersed in alcohol; F, the mold is secured with screws, nuts, and G-clamps after pouring the mixed silicone inside.

The mixture was poured into the mold, and the cover part was sealed over it. The mold was tightened by screws, nuts, and G-clamps. The mixture was left to set at (23°С ± 2°С) for 24 hours according to the manufacturer’s instructions. The specimens were stored at 20-25°C, 50 ± 10% humidity, and for 16 hours according to ISO 23529:2016 ( Figure 1F). ^15^

### Microbiological aspect of the study

*S. epidermidis* was isolated from three patients wearing maxillofacial prostheses who had skin lesions using sterile transport cotton swabs. By rotating the transport swab across the contaminated skin region, necrotic tissue was avoided. ^16^ It was then inoculated into blood agar and mannitol salt agar prepared according to the manufacturer’s instructions in an aerobic condition at 37 °C for 48 hours ( Figure 1A and B). ^17^ Identification of *S. epidermidis*: they form grayish-white, elevated, round, smooth, cohesive, 1–2 mm in diameter non-hemolytic colonies. They showed positive results in the catalyst test, and bacterial species were verified using the VITEK 2 compact identification system.

### Testing procedure

**Bacterial adherence test**

Biofilm formation on the silicone material was confirmed prior to the testing of its antibacterial efficacy using a scanning electron microscope. This test was performed to evaluate the antibacterial activity of BaTiO _3_ against *S. epidermidis*, depending on optical density (OD) measurement using a spectrophotometer (APEL PD-303, Japan) set at 600 nm. ^18^ Brain heart infusion broth was used to grow and create the bacterial suspension. It was prepared according to the manufacturer’s instructions by suspending 34.5 grams of powder in one liter of distilled water and dissolving it completely, then autoclaved at 15 lbs. of pressure (121°C) for 15 minutes. Then a suspension of 10 ^7^ colony forming units (CFU/ml) (0.5 McFarland standards) was prepared using a McFarland densitometer. The silicone specimens were sterilized for 20 minutes in an autoclave at 121°C. The sterile silicone specimens were placed in a sterile plastic dish containing the produced bacterial solution and incubated at room temperature for one hour ( Figure 1D). ^11^ Following completion of the incubation time, the specimens were withdrawn from the suspension, rinsed twice with phosphate-buffered saline for one minute with gentle rocking to remove any non-adherent bacterial cells, and dried on filter paper. ^11^ The specimens were then stained by 1% crystal violet for 10 minutes and rinsed well in phosphate-buffered saline ( Figure 1C). ^19^ Each specimen was immersed in 3 ml of 96% ethanol alcohol for 3 minutes; this solution was then used to confirm the optical density of each specimen ( Figure 1E). ^20^

**Fourier transforms infrared spectroscopy (FTIR)**

FTIR (IRAffinity-1 laser product, Shimadzu, Japan) was utilized to verify if silicone material and the BaTiO _3_ nanoparticles interacted chemically. Three samples, one from each group, were examined. (Control, 0.5wt% and 0.75wt%). The resolution was at 400-4000 cm ^-1^.

**Field emission scanning electron microscope (FE-SEM)**

The scattering of BaTiO _3_ nanoparticles within the silicone specimen matrix was evaluated using a FE-SEM (FEI, Netherland) machine. Three samples were tested, one from each group (control, 0.5wt% and 0.75wt%). According to the FE-SEM device used, the sample was prepared as follows: Cutting small pieces with scissors from the sample and coating them with gold using a sputter coater device for 2 minutes. The magnification was 1000, 4000, 13000, 25000, and 50000.

### The statistical analysis

The statistical analysis was performed using one-way ANOVA (analysis of variance) and post hoc tests (Games-Howell) by statistical analysis software (IBM SPSS Statistics 23, a proprietary free alternative we can suggest is PSPP). The Shapiro-Wilk test was used to discover the normality distribution of data, and Levene’s test was used to discover if the variances were homogenous.

The probability ( *P*) value was considered non-significant statistically (NS) when ( *P* > 0.05), while *P* value was considered statistically significant (S) when ( *P* ≤ 0.05), and *P* value was considered highly significant (HS) when ( *P* ≤ 0.01).

The sample size was estimated from previous studies. ^11^^,^^21^ Additionally, statistically, the minimum sample size can be 7 to 10 to be acceptable.

## Results

**Figure 2.**
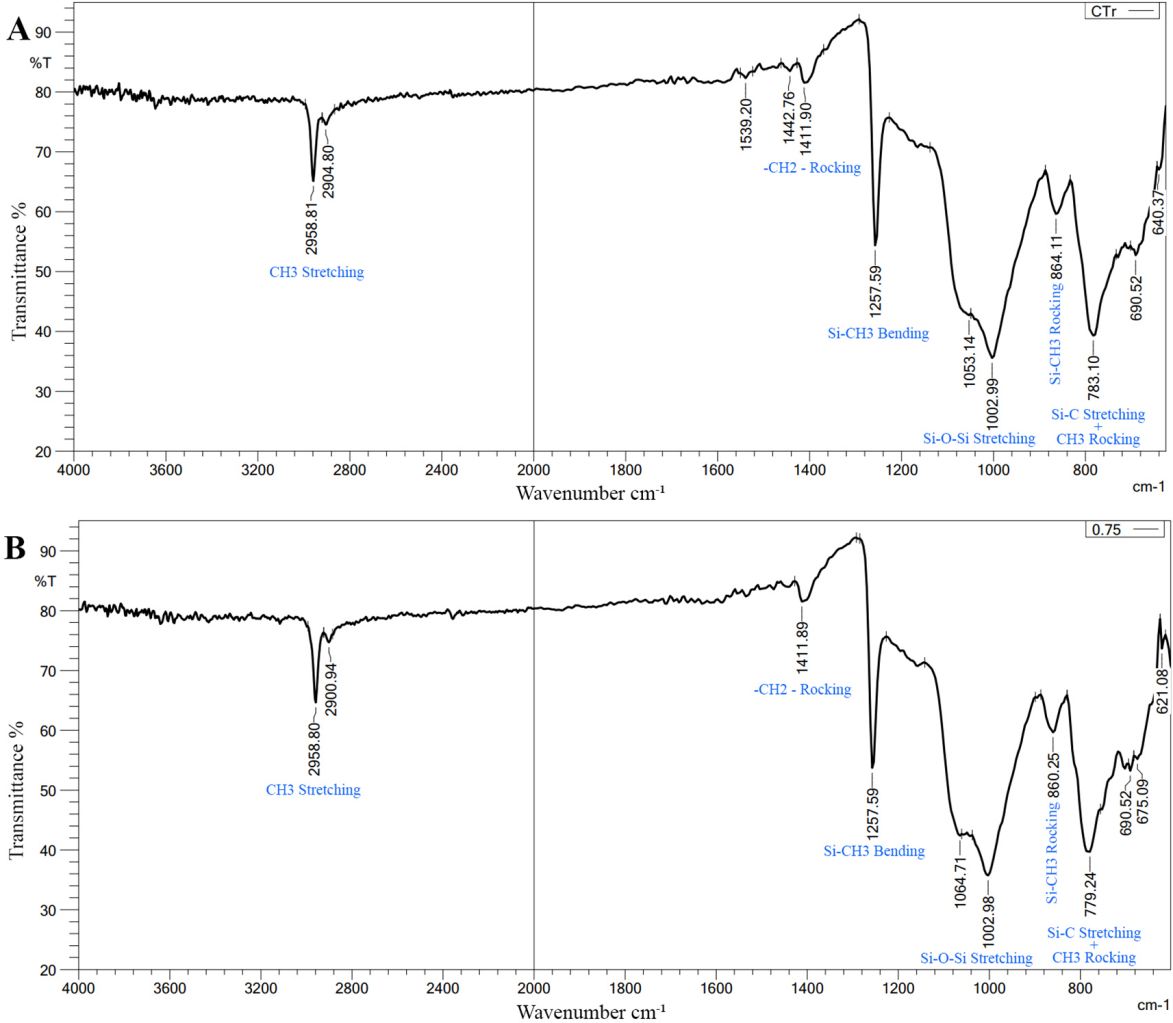
A, FTIR of control specimen; B, FTIR of 0.75wt% BaTiO _3_ specimen, revealing there is no chemical interaction. X axis refers to Wavenumber cm ^**-1**^, Y axis refers to Transmittance %. The bonds presented in the figure correspond to its peak wavenumber (blue font).

FTIR Results: There was no change in the spectra range of VST-50 silicone by the incorporation of BaTiO _3_ (no chemical interaction) as shown in Figure 2. The FTIR was used only to confirm if there was a chemical reaction or not, evaluating one test sample was enough to compare it with the control group.

**Figure 3.**
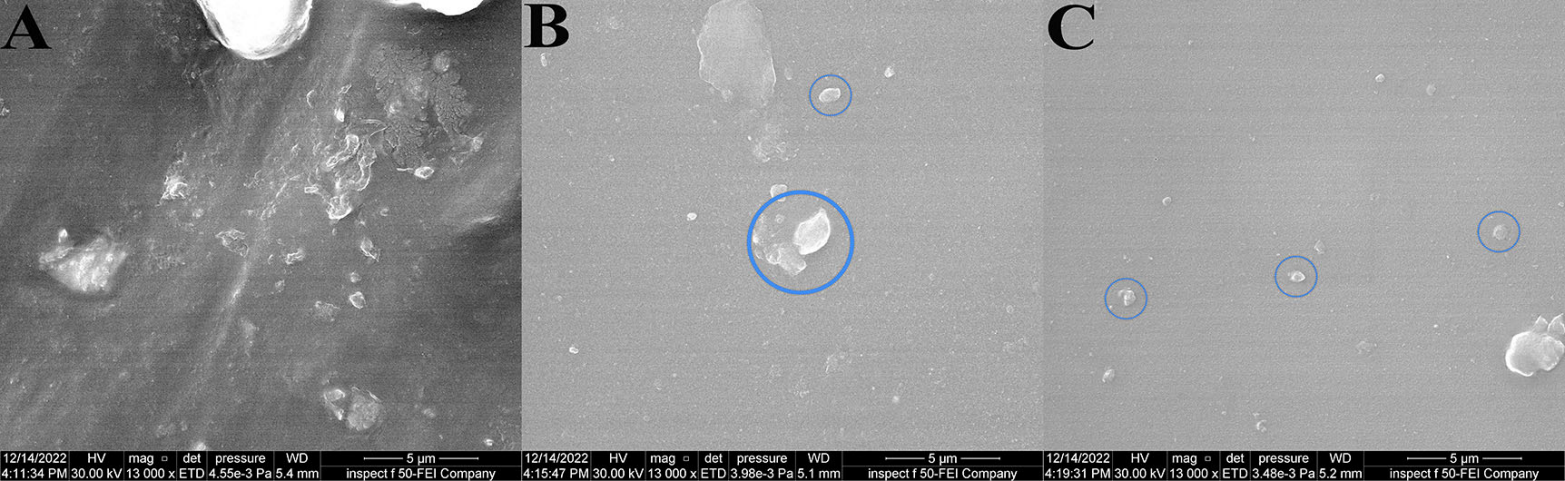
FE-SEM images at 13000 magnifications (5 μm) revealing evenly distribution of filler with a slight agglomerates as filler loading increases: A, control specimen; B, 0.5wt% specimens; C, 0.75wt% specimen. Circles show a number of large sized agglomerates of BaTiO _3_ as filler loading increases.

FE-SEM result: the BaTiO _3_ nanoparticles were evenly distributed throughout the VST-50 silicone matrix in the FE-SEM images, with slight agglomeration as filler loading increased, as shown in Figure 3. FE-SEM showed reduced silicone porosity.

### Bacterial adherence test result

The Shapiro-Wilk test revealed a normal distribution of data around the mean ( *P* value ˃ 0.05) ( Table 1).

**Table 1.** Shapiro-Wilk test of normality.

Shapiro-Wilk test			
Group	Statistic	df	Sig.
Control	0.974	10	0.923
0.25wt%	0.921	10	0.368
0.5wt%	0.937	10	0.520
0.75wt%	0.940	10	0.550
1wt%	0.848	10	0.055

**Figure 4.**
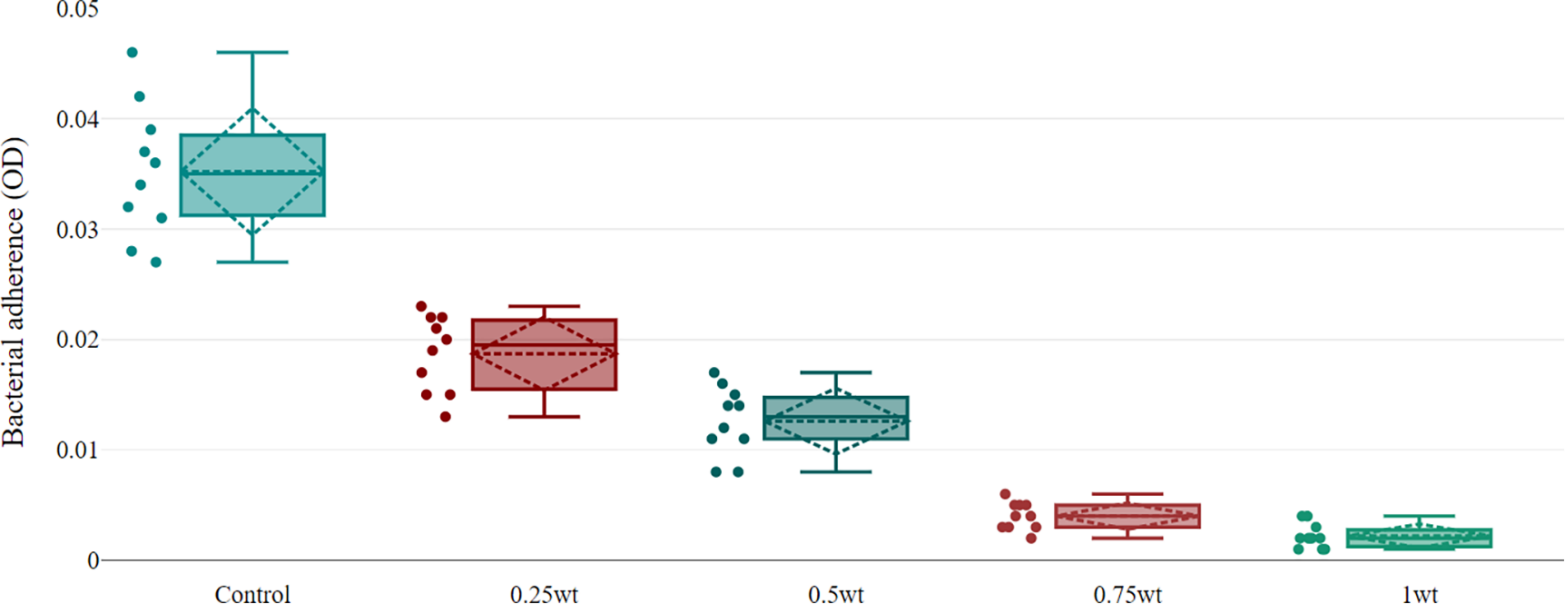
Boxplot shows maximum value (upper T-shaped whiskers), minimum value (lower T-shaped whiskers), mean (dashed line inside box), median (solid line inside box), standard deviation (dashed rhombic), and all conducted values (dots). The box indicates the range in which the middle 50% of all data, the lower end of the box is the 1st quartile and the upper end is the 3rd quartile, between q1 and q3 the interquartile range.

The descriptive statistic revealed a decrease in the mean of optical density (OD) as the concentration of BaTiO _3_ increased, which represented a decrease in bacterial adhesion ( Table 2), as shown in Figure 4.

**Table 2.** Descriptive statistics of bacterial adherence test (OD).

Group	No.	Mean	± SD	± SE	95% Confidence Interval for Mean		Min.	Max.
					Lower Bound	Upper Bound		
Control	10	0.0352	0.0060516	0.0019137	0.030871	0.039529	0.027	0.046
0.25wt%	10	0.0187	0.0034976	0.0011060	0.016198	0.021202	0.013	0.023
0.5wt%	10	0.0126	0.0031340	0.0009911	0.010358	0.014842	0.008	0.017
0.75wt%	10	0.004	0.0012472	0.0003944	0.003108	0.004892	0.002	0.006
1wt%	10	0.0022	0.0011353	0.0003590	0.001388	0.003012	0.001	0.004
Total	50	0.01454	0.0125083	0.0017689	0.010985	0.018095	0.001	0.046

One-way ANOVA test revealed a highly significant difference in the mean values among all groups ( *P* < 0.01) ( Table 3).

**Table 3.** One-way ANOVA analysis of variance among groups.

	Sum of Squares	df	Mean Square	F	*P* value
Between groups	0.007	4	0.002	144.515	0.000 HS
Within groups	0.001	45	0.000		
Total	0.008	49			

To choose the type of multiple comparison post hoc test and assess the homogeneity of variances, Levene’s test was used ( Table 4).

**Table 4.** Levene’s test for the bacterial adherence test.

Levene Statistic	df1	df2	*P* value	Sig.
7.762	4	45	0.000	S

Games-Howell test, revealed a highly significant difference between groups ( *P* < 0.01). except there was a significant difference between 0.75wt% group and 1wt% group at ( *P* < 0.05) ( Table 5).

**Table 5.** Games-Howell test of bacterial adherence test between groups.

Groups		Mean Difference	*P* value	Sig.
Control	0.25wt%	0.0165	0.000	HS
	0.5wt%	0.0226	0.000	HS
	0.75wt%	0.0312	0.000	HS
	1wt%	0.0330	0.000	HS
0.25wt%	0.5wt%	0.0061	0.005	HS
	0.75wt%	0.0147	0.000	HS
	1wt%	0.0165	0.000	HS
05wt%	0.75wt%	0.0086	0.000	HS
	1wt%	0.0104	0.000	HS
0.75wt%	1wt%	0.0018	0.025	S

## Discussion

Long-term usage of maxillofacial prostheses encourages the colonization of microorganisms on the silicone surface and spreads infection to nearby tissues; similarly, biofilm may transfer from infected skin to the prosthesis. ^5^

As previously stated, prolonged physical and chemical immersion disinfectants may result in material deterioration and color change, and the removal of bacterial accumulation is essential for external prostheses.

Additionally, it’s essential to discover a cleaning technique that is both effective in preventing infections and silicone prosthesis degeneration. ^22^ Due to the potential for a toxic or adverse effect, the use of any antimicrobial must be limited. The development of bacterial antibiotic resistance is one of the most urgent problems facing worldwide health care. In recent years, due to fewer side effects and effective antimicrobial activity, the use of oxides instead of chemical or synthetic medicine has increased. ^23^^–^^25^

In this investigation, it was shown to have an antibacterial action against the aforementioned bacteria since, as shown in Table 2, the percentages of bacterial cells adhering to the silicone specimens were dramatically reduced when compared to the control group.

Result of this study agreed with, ^10^ as they found a long-term antibacterial effect of BaTiO _3_ against *S.* *epidermidis* at 24 hours when added to Polyvinylsiloxane (PVS) between 5% and 15%. They also stated that the antibacterial activity was due to the release of Ba ^2 **+**^ and the formation of TiO _2_, resulting in slightly acidic environments. Then, when Ba ^2 **+**^ and TiO _2_ interact with water, they both help to create hydroxyl radicals (OH) and free radicals (O ^2 **-**^) that destroy nucleic acids, bacterial cell walls, and other molecular structures.

Swain *et al*. found that the positively charged hydroxyapatite-BaTiO _3_ composite revealed antibacterial activity against *S. aureus*, *E. coli*, and *P. aeruginosa* with a remarkable inhibition zone. Positively polarized HA-BT composites rupture the bacterial membrane in vitro. ^26^ Additionally, many studies have shown that barium titanate has antifungal activity. ^27^^,^^28^

FTIR measurements were performed both prior to and following the addition of BaTiO _3_ nanoparticles. As the spectral range remained unchanged both prior to and following the addition, there was no chemical reaction. The only interaction in this case is described as a physical reaction (hydrogen bond or Van der Waals bond), and it results from fillers interacting with silicone. This interaction manifested as a slight change in the vibration of preexisting bonds and a change in the silicone matrix’s light transmittance. This confirms that the antibacterial activity is related to BaTiO _3_, as no new chemical material was produced, and explains the difference in antibacterial activity between the control and experimental groups.

FE-SEM revealed well dispersion of BaTiO _3_ inside the silicone matrix with some agglomeration as the filler percentage increased, and this agreed with. ^1^^,^^29^ And disagreed with other studies because they utilized different fillers in varying quantities and agglomeration was only noticeable at higher percentages. This could be because surface-treated silicon dioxide nanoparticles were used; surface treatment impacts the dispersion of the nanofiller inside the matrix by decreasing the probability of nanoparticle aggregation. ^30^^,^^31^

Another factor that affected the reduction in bacterial adhesion was reduced porosity. Many studies confirm that the addition of non-filler materials to various materials reduces porosity since the filler fills the space inside the matrix of materials. ^32^^,^^33^ FE-SEM results showed reduced porosity of the silicone matrix, which reduces the opportunity for bacterial adhesion.

## Conclusions

With respect to the limitations of this study, it can be concluded that the addition of BaTiO _3_ powder to VST-50 maxillofacial silicon elastomer will enhance the antibacterial activity of silicon against *Staphylococcus epidermidis*, and this activity seems to be concentration dependent. For further study we could evaluate the effect of the addition of BaTiO _**3**_ nanoparticles on the fungal biofilm’s adhesion to the maxillofacial silicones and study the effects of adding BaTiO _**3**_ nanoparticles to pigmented VST-50 RTV silicone elastomers. Evaluating the artificial aging of VST-50 RTV maxillofacial silicone after the addition of BaTiO _**3**_ nanopowder is another suggestion that could be explored.

## Data Availability

Figshare. Antibacterial effect of Barium Titanate, https://doi.org/10.6084/m9.figshare.22336786.v1. ^34^ This project contains the following underlying data:
• Raw data. (optical density of bacterial test) • FTIR data. (for BaTiO _3_ and for silicone before and after addition of BaTiO _3_) • FE-SEM data (pictures for BaTiO _3_ and for silicone before and after addition of BaTiO _3_) • VITEK 2 Microbiology Chart Report • Pictures of steps of bacterial test • Particle size analyzer report of barium titanate Data are available under the terms of the Creative Commons Attribution 4.0 International license (CC-BY 4.0).
