# Supplementary material for: Assessment of the antibacterial effect of Barium Titanate nanoparticles against  Staphylococcus epidermidis adhesion after addition to maxillofacial silicone
Source: F1000Res. 2023 Nov 6;12:385. Originally published 2023 Apr 12. [Version 2] doi: 10.12688/f1000research.132727.2 (PMC10468664; doi:10.12688/f1000research.132727.2)
Supplement: Peer review history

# Reviewer report: Reviewer response for version 2

**DOI:** 10.5256/f1000research.158425.r221087

**Published:** 2023-11-10

**Authors:** Nidambur Vasudev Ballal

**Affiliations:** 1 Department of Conservative Dentistry and Endodontics, Manipal College of Dental Sciences, Manipal Academy of Higher Education, Manipal, Karnataka, India

The authors have revised their manuscript appropriately based on referee comments. Hence, the manuscript can be approved now for indexing.

 Thank you

Is the work clearly and accurately presented and does it cite the current literature?

Yes

If applicable, is the statistical analysis and its interpretation appropriate?

Yes

Are all the source data underlying the results available to ensure full reproducibility?

Yes

Is the study design appropriate and is the work technically sound?

Partly

Are the conclusions drawn adequately supported by the results?

Yes

Are sufficient details of methods and analysis provided to allow replication by others?

Partly

Reviewer Expertise:

Microbiology

I confirm that I have read this submission and believe that I have an appropriate level of expertise to confirm that it is of an acceptable scientific standard.

# Reviewer report: Reviewer response for version 1

**DOI:** 10.5256/f1000research.145673.r201564

**Published:** 2023-09-12

**Authors:** Nidambur Vasudev Ballal

**Affiliations:** 1 Department of Conservative Dentistry and Endodontics, Manipal College of Dental Sciences, Manipal Academy of Higher Education, Manipal, Karnataka, India

This manuscript constitutes an attempt to assess the antibacterial effect of Barium Titanate nanoparticles against *Staphylococcus epidermidis *adhesion after incorporation to maxillofacial silicone. The study is relevant to the journal and well performed. Some of the specific queries have been addressed below.

**Introduction:**

- Mention the rationale of performing this study. There are several antibacterial nanoparticles like Silver, Chitosan, Zinc, Titanium etc. which have incorporated into silicone maxilla-facial prosthesis to exhibit antibacterial activity (Chong *et al.,* (2022) ^1^; Cevik *et al.,* (2023) ^2^). Why specifically, Barium Titanate nano-particles were tested in this study? Mention in detail.
- Mention the hypothesis of the study tested.

**Methodology:**

- A positive control group of chlorhexidine should have been used.
- How was sample size estimated?
- Why in control group, was silicone material mixed using digital weighing balance when compared to test groups which were mixed with vacuum pressure?
- Did the skin of the patient from which the swab obtained have any lesions or disease? If so, mention it.
- How was biofilm formation on the silicone material confirmed prior to the testing of antibacterial efficacy of test agents?
- In FTIR analysis, mention the resolutions at which the spectra were obtained.
- In FESEM analysis, mention how was samples prepared for the analysis and also at what magnification the images were captured.
- In FTIR and FESEM analysis, why samples treated with 0.25 and 1% BaTiO3 were not evaluated?

**Results:**

- In FESEM image, in control group, what are those particles seen? Also, how was the even distribution of the filler particles assessed? In test group, only few filler particles are seen.
- In FTIR and FESEM analysis, why statistics was not performed? Was it only qualitative data obtained?

Is the work clearly and accurately presented and does it cite the current literature?

Yes

If applicable, is the statistical analysis and its interpretation appropriate?

Yes

Are all the source data underlying the results available to ensure full reproducibility?

Yes

Is the study design appropriate and is the work technically sound?

Partly

Are the conclusions drawn adequately supported by the results?

Yes

Are sufficient details of methods and analysis provided to allow replication by others?

Partly

Reviewer Expertise:

Microbiology

I confirm that I have read this submission and believe that I have an appropriate level of expertise to confirm that it is of an acceptable scientific standard, however I have significant reservations, as outlined above.

## References

[1] : Efficacy of incorporating silver nanoparticles into maxillofacial silicone against Staphylococcus aureus, Candida albicans, and polymicrobial biofilms. *J Prosthet Dent* .2022;128(5) : 10.1016/j.prosdent.2021.01.010 1114-1120 10.1016/j.prosdent.2021.01.010 33685653

[2] : Antimicrobial effects of nano titanium dioxide and disinfectants on maxillofacial silicones. *J Prosthet Dent* .2023; 10.1016/j.prosdent.2023.03.001 10.1016/j.prosdent.2023.03.001 37012133

# Response

**Published:** 2023-10-15

**Authors:** Yasir Mohammed Kareem

**Affiliations:** prosthodontic, University of Baghdad, Baghdad, Baghdad Governorate, Iraq

Dear Nidambur Vasudev Balla,

 Thank you for taking the time to review our article titled **“Assessment of the antibacterial effect of Barium Titanate nanoparticles against *Staphylococcus epidermidis* adhesion after addition to maxillofacial silicone” **and providing us with your insightful feedback to improve the manuscript. We have included answers in the manuscript to the questions put forth. Please see below the point-by-point responses to the questions.

**Introduction:**

 "Mention the rationale of performing this study. There are several antibacterial nanoparticles like Silver, Chitosan, Zinc, Titanium etc. which have incorporated into silicone maxilla-facial prosthesis to exhibit antibacterial activity (Chong *et al.,* (2022) ^1^ ; Cevik *et al.,* (2023) ^2^ ). Why specifically, Barium Titanate nano-particles were tested in this study? Mention in detail."

 Response: Thank you for your comment, As mentioned in the introduction in the fourth paragraph "the primary cause of routine facial prosthetic replacement is deterioration in appearance caused by changes in physical characteristics and color". So not only the antibacterial effect of BaTiO _3_ is important. The other mechanical and physical properties of BaTiO _3_ absolutely differ from those of silver, chitosan, zinc, titanium, etc. This may be superior or inferior. This study can be followed by other studies to evaluate the mechanical and physical properties of BaTiO _3_, as mentioned in the suggestion.

 "Mention the hypothesis of the study tested."

 Response: 

**The null hypothesis (H0):** Suggested that adding BaTiO **_3_** Nanoparticles will not affect bacterial adherence.

**The alternative hypothesis (H1):** Suggested that adding BaTiO **_3_** Nanoparticles will significantly reduce bacterial adherence.

**Methodology:**

 "A positive control group of chlorhexidine should have been used."

 Response: Chlorhexidine is antibacterial, has been approved in many studies, and needs no more approval. Additionally, we need to discover the effects of BaTiO _3_ on the physical and mechanical properties of silicon, not only its antibacterial activity. For this reason, we compared the silicone without a positive control. If the antibacterial property is the only property that we need, we would use chlorhexidine as a positive control to compare the antibacterial activity of BaTiO _3_ with it.

 "How was sample size estimated?"

 Response: The sample size estimated from previous studies, such as

- Ibrahim HI, Abdul-Ameer FM: Influence of kappa-carrageenan powder addition on staphylococcus epidermidis adhesion on the room-temperature vulcanized maxillofacial silicone. *Pak. J. Med. Health Sci.* 2021; **15** : 359

Additionally, statistically, the minimum sample size can be 7 to 10 to be acceptable.

 "Why in control group, was silicone material mixed using digital weighing balance when compared to test groups which were mixed with vacuum pressure?"

 Response: The digital balance was used for all groups (control and test) and only for weighing the components that would be mixed. and the vacuum mixer was also used for all groups (control and test) to avoid air entrapment. The weighing and mixing for all groups were done in the same way. 

 I am sorry, but you may have had a misunderstanding while reading the mixing part.

 "Did the skin of the patient from which the swab obtained have any lesions or disease? If so, mention it."

 Response: Yes, the skin patients have a lesion, and they had a facial prosthesis.

 "How was biofilm formation on the silicone material confirmed prior to the testing of antibacterial efficacy of test agents?"

 Response: It was confirmed using scanning electron microscope.

 "In FTIR analysis, mention the resolutions at which the spectra were obtained."

 Response: the resolutions was at 400-4000 cm ^-1^

 "In FESEM analysis, mention how was samples prepared for the analysis and also at what magnification the images were captured."

 Response: According to the FE-SEM device used, the sample was prepared as follows: Cutting small pieces with scissors from the sample and coating them with gold using a sputter coater device for 2 minutes. The magnification was 1000, 4000, 13000, 25000, and 50000.

 "In FTIR and FESEM analysis, why samples treated with 0.25 and 1% BaTiO3 were not evaluated?"

 Response: The FTIR and FE-SEM were used to obtain qualitative data.

 Because the FTIR was used only to confirm if there was a chemical reaction or not, evaluating one test sample was enough to compare it with the control group.

 Also, FE-SEM was used to evaluate the distribution of BaTIO3 in the silicon matrix, and we took two different percentages. and they were enough to show the distribution of filler and some agglomeration as filler concentration increased.

**Results:**

**"**In FESEM image, in control group, what are those particles seen? Also, how was the even distribution of the filler particles assessed? In test group, only few filler particles are seen."

 Response: These particles are sputter coating materials related to the device, and they are seen only at low magnifications. and some surface roughness was also seen.

 the even distribution of filler assessed by a observation

 The even distribution of filler was assessed by observation. It seems uniform, and in some areas there are some agglomerations.

 Your comment that "only a few filler particles are seen" could be due to the very tinny size of the nanoparticles (59.4 nm). and could appear at greater magnifications. These few particles could be a cluster of more than one particle. and the white, tiny particles are the single ones.

 "In FTIR and FESEM analysis, why statistics was not performed? Was it only qualitative data obtained?"

 Response: Yes, it was only qualitative data obtained to confirm the type of reaction and distribution of filler.

 Finally, we thank you again for your valuable and insightful comments.

# Reviewer report: Reviewer response for version 1

**DOI:** 10.5256/f1000research.145673.r184170

**Published:** 2023-08-30

**Authors:** Kadhim A. Hubeatir

**Affiliations:** 1 Department of Laser and Optoelectronics Engineering, University of Technology, Baghdad, Baghdad, Iraq

I think this research was an experimental and revealed a new results in dentistry especially to enhance the prevention and to improve the efficiency of dentin against antibacterial effect using a Barium Titanate nanoparticles.

 A good statistically analysis using ANOVA method and another different methods which was give a highly significant difference between them. Also FTIR analysis shows no chemical reactions between the Barium Titanate and Maxillofacial silicon elastomer. The pictures of SEM shows the nano powder of barium titanate dispersed inside the maxillofacial silicon.

Is the work clearly and accurately presented and does it cite the current literature?

Yes

If applicable, is the statistical analysis and its interpretation appropriate?

Yes

Are all the source data underlying the results available to ensure full reproducibility?

Yes

Is the study design appropriate and is the work technically sound?

Yes

Are the conclusions drawn adequately supported by the results?

Yes

Are sufficient details of methods and analysis provided to allow replication by others?

Yes

Reviewer Expertise:

Material science and Nanotechnology, Laser application in medicine specially in dentistry

I confirm that I have read this submission and believe that I have an appropriate level of expertise to confirm that it is of an acceptable scientific standard.

# Reviewer report: Reviewer response for version 1

**DOI:** 10.5256/f1000research.145673.r192100

**Published:** 2023-08-30

**Authors:** Razia Z. Adam

**Affiliations:** 1 University of the Western Cape, Cape Town, South Africa

This article addresses the application of nanoparticles to improve the properties of maxillofacial silicone. Barium Titanate is explored as a potential modifier which will improve the antibacterial effect of the silicone. Prepared nps are tested against S Epidermidis specifically with five groups including a control group. Characterization test confirm the nps and SEM also clearly indicates the dispersion in the material. Antibacterial activity was tested and yielded promising results.

 The Methodology is well explained in detail. The results and discussion are appropriate.

Is the work clearly and accurately presented and does it cite the current literature?

Yes

If applicable, is the statistical analysis and its interpretation appropriate?

I cannot comment. A qualified statistician is required.

Are all the source data underlying the results available to ensure full reproducibility?

Yes

Is the study design appropriate and is the work technically sound?

Yes

Are the conclusions drawn adequately supported by the results?

Yes

Are sufficient details of methods and analysis provided to allow replication by others?

Yes

Reviewer Expertise:

Dental biomaterials, application of nps

I confirm that I have read this submission and believe that I have an appropriate level of expertise to confirm that it is of an acceptable scientific standard.
